# Diagnostic accuracy and microbial profiles of tuberculous pleurisy: a comparative study of metagenomic next generation sequencing and GeneXpert *Mycobacterium tuberculosis*

**DOI:** 10.3389/fcimb.2023.1243441

**Published:** 2023-11-27

**Authors:** Fengxiang Huang, Haoran Wang, Ruiping Qiao, Qiang Peng, Chang Zhao, Lijun Miao

**Affiliations:** ^1^ Department of Respiratory and Critical Care Medicine, First Affiliated Hospital of Zhengzhou University, Zhengzhou, China; ^2^ Department of Respiratory and Critical Care Medicine, Chest Hospital of Henan Province, Zhengzhou, China

**Keywords:** metagenomic next-generation sequencing, tuberculous pleurisy, pleural effusion, *Mycobacterium tuberculosis*, diagnostic techniques and procedures, sensitivity and specificity

## Abstract

**Introduction:**

There is a clinical challenge in diagnosing tuberculous pleurisy accurately and promptly, highlighting the urgent need for a rapid and sensitive diagnostic method. This study aimed to evaluate the diagnostic accuracy of metagenomic next-generation sequencing (mNGS) and GeneXpert *Mycobacterium tuberculosis* (MTB) for identifying tuberculous pleurisy and analyzing the microbial profiles of both tuberculous and non-tuberculous pleural effusions.

**Methods:**

The study enrolled 31 patients with suspected tuberculous pleurisy, of which 15 were confirmed to have tuberculous pleurisy and subsequently allocated to the tuberculous pleurisy group (TP group), while the remaining 16 individuals were assigned to the non-tuberculous pleurisy group (NTP group). mNGS and GeneXpert MTB were performed on pleural effusion samples, and the diagnostic accuracy of both tests was compared. We employed established formulas to compute crucial indicators, including sensitivity, specificity, missed diagnosis rate, misdiagnosed rate, positive predictive value (PPV), and negative predictive value (NPV).

**Results:**

The results showed that both tests had high specificity (100%) and positive predictive value (100%) for detecting tuberculous pleurisy, along with comparable sensitivity (46.67% for mNGS and 40.0% for GeneXpert MTB). Further analysis of the combined efficacy of mNGS and GeneXpert MTB showed that the combined test had a sensitivity of 66.67% and a specificity of 100%. mNGS analysis revealed that MTB was detected in 7 out of 15 patients with tuberculous pleural effusions, while non-tuberculous pleural effusions were associated with a diverse range of microbial genera and species. The most frequently detected genera at the microbial genus level in the NTP group were *Microbacterium* spp. (6/16), *Prevotella* spp. (5/16), and *Campylobacter* spp. (5/16).

**Discussion:**

These findings suggest that mNGS and GeneXpert MTB are useful diagnostic tools for identifying patients with tuberculous pleurisy, and mNGS can provide valuable insights into the microbial profiles of both tuberculous and non-tuberculous pleural effusions.

## 1 Introduction

Tuberculous pleurisy is a significant cause of death worldwide, imposing a huge medical burden as the most common form of extrapulmonary tuberculosis (TB) (WHO, 2021). Approximately 50% of extrapulmonary TB patients are diagnosed with tuberculous pleurisy (Kang et al., 2020), which is characterized by a severe immune response with high exudation of adenosine deaminase (ADA), lymphocyte enrichment, and local exudation of neutrophils (Shaw et al., 2018). The disease can lead to serious complications, such as pleural thickening or adhesions, tuberculous empyema, and bronchopleural fistula, which can severely impact the patient’s health and quality of life if not detected and treated promptly (Barbas et al., 1991; Sonmezoglu et al., 2008; Shaw et al., 2019).

The current diagnostic methods for tuberculous pleurisy include imaging, sputum smear microscopy, *mycobacterium tuberculosis* (MTB) culture, MTB nucleic acid testing, pleural biopsy, and immunologic testing (Ferreiro et al., 2020). The MTB culture is widely regarded as the gold standard, however, the process is time-consuming, typically requiring 4-8 weeks. Additionally, it exhibits limited sensitivity. The sensitivity in pleural effusion, as reported by various literature, ranges from 20% to 40% (Gopi et al., 2007; Light, 2010; Shaw et al., 2019). The diagnostic accuracy of pleural tissue biopsy for tuberculous pleurisy is substantial, reaching 69%-97%. This can even achieve 100% sensitivity through thoracoscopic biopsy, and it’s even higher in HIV-positive individuals (Lewinsohn et al., 2017; Casalini et al., 2018). Despite its high accuracy, pleural tissue biopsy is invasive and technically challenging, often accompanied by potential risks, rendering it unavailable in certain situations. Although immunological diagnostic methods such as ADA, interferon-γ, and interleukin-27 in pleural effusion are simple and fast, their accuracy is limited (Mollo et al., 2017; Lo Cascio et al., 2021). Recently, molecular diagnostic technology has brought breakthroughs to the diagnosis of tuberculous pleurisy, with GeneXpert MTB nucleic acid amplification technology (GeneXpert MTB) being recommended by WHO for confirming pulmonary and suspected extrapulmonary tuberculosis patients (Nhu et al., 2014). However, its sensitivity, particularly in extrapulmonary TB, remains low, and it can only target specific mycobacterial antigens or antibodies of known gene sequences. In addition, the lack of MTB in pleural fluid is also a great challenge for ordinary PCR detection methods to capture MTB information (Antonangelo et al., 2019). Therefore, there is a need to develop rapid, convenient, and accurate diagnostic methods that can facilitate the timely and precise diagnosis and treatment of tuberculous pleurisy.

Metagenomic next-generation sequencing (mNGS) is a powerful sequencing technique that can simultaneously sequence thousands to billions of DNA fragments, enabling the detection of nucleic acid sequence information for all species present in a sample, without being dependent on bacterial culture or antigen/antibody levels (Simner et al., 2018). This technique is fast, convenient, and has a wide detection range, making it particularly useful for detecting rare, novel, and unknown pathogen sequences. mNGS has been successfully used to diagnose pathogen infections in various sites, such as the central nervous system, lungs, blood, skin, and abdomen (Gu et al., 2019). In addition, mNGS can also be used for microbiome studies of pulmonary diseases. Research has shown that tuberculosis patients have a complex microbial environment, and there are differences in microbial flora composition and distribution between healthy individuals and tuberculosis patients, which may affect the development and treatment of the disease (Hong et al., 2018; Hu et al., 2020; Xiao et al., 2022). However, the diagnostic value of mNGS in tuberculous pleurisy has not been fully established yet.

The purpose of the study was to evaluate the diagnostic accuracy and efficacy of mNGS and GeneXpert MTB in the diagnosis of tuberculous pleurisy and to analyze the microbial profiles of tuberculous and non-tuberculous pleural effusions using mNGS. Our results may help improve the accuracy of diagnosis for tuberculous pleurisy, which can aid in the timely initiation of appropriate treatment and ultimately improve patient outcomes.

## 2 Materials and methods

### 2.1 Patients and sample collection

This study was approved by The Ethics Committee of The First Affiliated Hospital of Zhengzhou University (approval number: 2022-KY-1276-002). A total of 31 patients with suspected tuberculous pleurisy who visited The First Affiliated Hospital of Zhengzhou University and Chest Hospital of Henan Province Chest Hospital between September 2021 and September 2022 were included in this study. Pleural effusion samples were collected for mNGS and GeneXpert MTB detection. The inclusion criteria were as follows: 1) participants must be at least 18 years old; 2) participants must be suspected of having tuberculous pleurisy, with clinical symptoms related to pleurisy such as fever, cough, chest pain, and dyspnea, and chest imaging suggesting pleural effusion; 3) complete clinical data must be available for participants; 4) specimens of pleural effusion must have been submitted for mNGS sequencing; 5) participants must have understood the purpose and significance of the study and voluntarily signed the informed consent form. The exclusion criteria were: 1) patients who have received anti-tuberculosis treatment before admission; 2) patients with underlying congenital or acquired immunodeficiency diseases; 3) pregnant or lactating patients; 4) mNGS sequencing results showing that the quality control did not meet the standard.

Confirmed tuberculous pleurisy was defined as a positive result for MTB detected by bacterial or molecular methods in sputum, bronchoalveolar lavage fluid (BALF), pleural effusion, lung or pleural tissue specimens, or the biopsy of lung or pleural showing caseous granuloma. Non-tuberculous pleurisy was defined as confirmation of a diagnosis other than tuberculosis through microbiology, histopathology, or serology examination. Based on the diagnostic criteria, patients were classified into two groups: the tuberculous pleurisy group (TP group) and the non-tuberculous pleurisy group (NTP group). Demographic and clinical data, including age, gender, symptoms, and laboratory and radiological results, were collected from their medical records (**Table 1**). Specimens were obtained through thoracentesis or closed drainage of the pleural cavity. Twenty milliliters of pleural fluid were collected using EDTA anticoagulation tubes and transported to the laboratory under refrigeration within 4 hours for subsequent mNGS and Genexpert MTB testing.

**Table 1:** Clinical characteristics of patients with tuberculous and non-tuberculous pleurisy.

Characteristics	TP (n)	NTP (n)
Age (years)		
≤ 30	5	0
31–60	9	8
≥ 61	1	8
Sex		
Male	14	14
Female	1	2
**Smoking history**	5	8
**Diabetes**	3	1

TP, tuberculous pleurisy; NTP, non-tuberculous pleurisy.

### 2.2 mNGS

Each pleural fluid sample (1.2 mL) was meticulously aspirated and transferred into a 2 mL shock tube. Subsequently, the shock tube underwent efficient disruption of cell walls by a sample shock breaker (BSP-100; Hangzhou Matridx Biotechnology Co., Ltd., China). Then it was centrifuged at 12,000 rpm for 3 minutes. 400 μL of supernatant was pipetted into the cartridge (MD013, Hangzhou Matridx Biotechnology Co., Ltd., China) with pre-loaded reagents. Finally, the cartridge was inserted into the device of NGSmasterTM (MAR002; Hangzhou Matridx Biotechnology Co., Ltd., China), which could automatically complete nucleic acid extraction, PCR-free library preparation (enzymatic fragmentation, end repairing, terminal adenylation and adaptor ligation) and purification. Finished libraries were quantified by real-time PCR (KAPA) and pooled for sequencing. Shotgun sequencing was carried out on illumina Next seq platform. Approximately 20 million of 75 bp single-end reads were generated for each library. Bioinformatic analysis was conducted. Briefly, after filtering out the sequences of human origin (hg38) and removing adaptors, the remaining reads were aligned to a reference database (NCBI Nt, Patric, ViPR, EupathDB, fungibank and in-house curated microbial genomic data) to identify microbial species, reads count and relative abundance. For each run, one negative control (artificial plasma mixed with fragmented human genomic DNA) was included for quality control. The test results of microbial species identified from a sample were reported according to the following criteria: (a) the results passed quality control filters (library concentration >50 pM, Q20 > 85%, Q30 > 80%); (b) negative control (NC) in the same sequencing run does not contain the species or the ratio of reads per million in NC (RPMNC) to reads per million in sample (RPMsample) is less than 5.

### 2.3 GeneXpert MTB

The pleural effusion samples were treated with 4% NaOH solution (NaOH solution to sample ratio of 2:1), followed by centrifugation at 3000 r/min and subsequent removal of the supernatant. The resulting centrifugal precipitate was resuspended in a 67mM PBS solution. Subsequently, the experimental procedure was conducted according to the Xpert MTB/RIF reaction kit (Cepheid, USA). Briefly, 1.5 ml of sample reagent (SR) was added to 0.5 ml of suspended sediment (SR to sample ratio of 3:1). The test tube containing SR and sample was vigorously shaken for 10-20 times (one back and forth movement constitutes a single shake) or vortexed, then incubated at room temperature for 10 minutes. After an additional vigorous shaking and incubation for 5 minutes, 2 ml of the material were transferred into the test cartridge which was inserted into the MTB-RIF test platform. An automated test then commenced including automatic filtration and washing of the sample, lysis of filter-captured organisms to release DNA, seminested real-time PCR amplification and detection. finally, the test result could be automatically printed within approximately 2 hours.

### 2.4 Statistical analysis

The enumeration data were reported as “number of cases” and “rate”. Values of sensitivity, specificity, misdiagnosis rate (false positive rate), missed diagnosis rate (false negative rate), positive predictive value (PPV), and negative predictive value (NPV) for both mNGS and GeneXpert MTB were calculated through established formulas. The differences between the two methods were compared using the Chi-square test and Fisher’s exact test through the SPSS 19.0 version software. A significance level of P value set at 0.05 guided these comparative assessments.

## 3 Results

### 3.1 Clinical characteristics of patients with suspected tuberculous pleurisy

A total of 31 patients, including 28 males and 3 females with a minimum age of 18 years old and a maximum age of 86 years old, were enrolled in this study due to suspected tuberculous pleurisy (**Table 1**). The majority of pleural effusions occurred on the right side (19 patients), followed by the left side (7 patients) and bilateral (5 patients). Common clinical symptoms included cough (13 cases), fever (12 cases), chest or back pain (12 cases), chest tightness (16 cases), and in rare cases, bloody phlegm (1 case) and dyspnea (1 case) were reported. Imaging tests revealed that pulmonary infiltration was characterized by exudation (patchy and floccus) in 17 cases, nodules in 9 cases, consolidation in 8 cases, fibro stripe shadows in 6 cases, atelectasis in 5 cases, mass shadows in 3 cases, and cavity in 3 cases.

**Figure 1:**
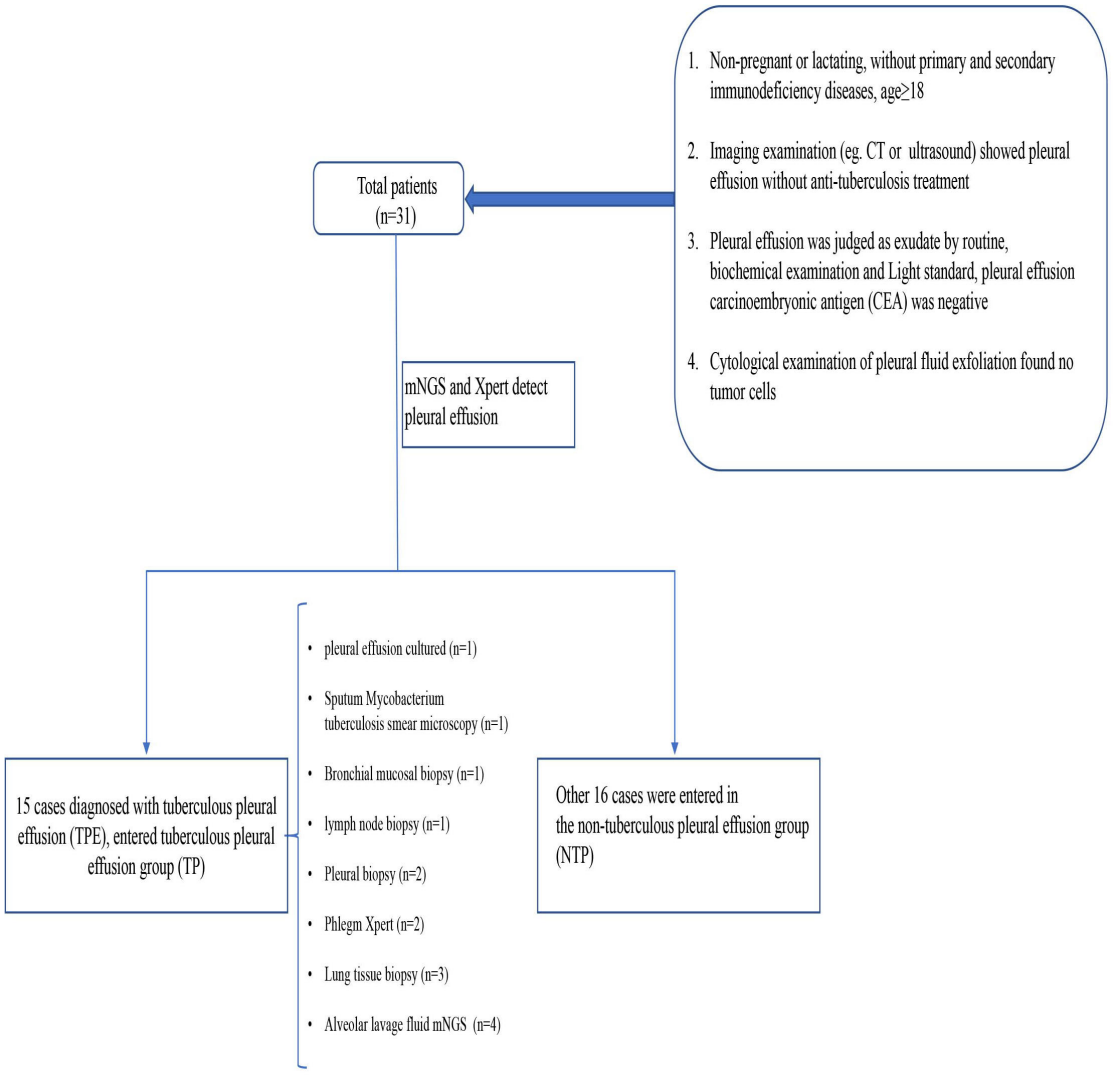
Flow chart of the study design.

Among these patients, 15 individuals were diagnosed with tuberculous pleurisy (**Figure 1**). The confirmation process unfolded as follows: One case was unequivocally confirmed through the detection of a positive pleural fluid culture, and one by a positive sputum smear microscopy. The diagnosis in two cases was corroborated by a positive sputum Xpert test. Importantly, mNGS detected the mycobacterium tuberculosis complex group in the bronchoalveolar lavage fluid (BALF) of four cases, further solidifying their diagnoses. An additional seven cases were substantiated through tissue biopsies, each providing unique perspectives for confirmation: one case found validation through a bronchial mucosal tissue biopsy; lymph node biopsy firmly established the diagnosis for one case; pleural biopsies were instrumental in confirming two cases; and finally, lung biopsy was utilized to confirm three other cases. 15 patients were assigned to TP group (cases 17–31 in **Table 2**), while the remaining 16 patients were assigned to NTP group (cases 1–16 in **Table 2**). More information can be found in **Figure 1** and **Table 2**.

**Table 2:** Patient characteristics and diagnostic results of tuberculous and non-tuberculous pleurisy cases.

Number	Sex	Age	Symptoms	Location	Pulmonary infiltration	mNGS results	GeneXpert results	Diagnosis
1	male	68	none	right	stripe shadows	negative	negative	NTP
2	male	56	cough/fever/ chest pain	left	patchy exudation / mass shadows	negative	negative	NTP
3	male	86	dyspnea	bilateral	floccus exudation	negative	negative	NTP
4	male	81	cough/chest tightness	bilateral	nodules/patchy exudation	negative	negative	NTP
5	male	75	cough/bloody phlegm/chest tightness	right	stripe shadows	negative	negative	NTP
6	male	63	fever/cough/ sputum/chest tightness	right	consolidation /atelectasis	negative	negative	NTP
7	male	49	chest pain	right	patchy exudation /nodules /atelectasis	negative	negative	NTP
8	male	56	fever	right	stripe shadows	negative	negative	NTP
9	male	77	chest tightness	right	nodules/cavity	negative	negative	NTP
10	male	52	fever/ chest pain	right	consolidation	negative	negative	NTP
11	female	58	chest tightness	right	consolidation /atelectasis	negative	negative	NTP
12	male	45	cough/ sputum/chest tightness	left	patchy exudation /small cavity	negative	negative	NTP
13	male	70	fever	right	patchy exudation	negative	negative	NTP
14	female	43	chest pain/chest tightness	right	patchy exudation /nodules	negative	negative	NTP
15	male	71	chest tightness	bilateral	stripe shadows	negative	negative	NTP
16	male	50	cough/chest tightness	right	patchy exudation	negative	negative	NTP
17	male	25	fever/cough/ chest pain	left	patchy exudation /nodules/local consolidation	positive	positive	TP
18	female	45	chest and back pain	right	patchy exudation /mass shadows	positive	negative	TP
19	male	50	cough/fever/ chest pain	right	patchy exudation /nodules/small cavity	negative	positive	TP
20	male	56	fever/chest and back pain /chest tightness	right	mass shadows	negative	negative	TP
21	male	32	fever/chest pain/chest tightness	left	consolidation /patchy exudation	negative	positive	TP
22	male	45	cough/chest tightness	right	none	positive	negative	TP
23	male	18	cough/sputum	left	patchy exudation /atelectasis	negative	negative	TP
24	male	56	cough/chest pain	right	patchy exudation	negative	negative	TP
25	male	18	chest pain	right	nodules/local consolidation	positive	negative	TP
26	male	33	fever/chest tightness	left	stripe shadows	positive	positive	TP
27	male	54	fever/chest tightneess	left	patchy exudation /nodules	positive	positive	TP
28	male	69	chest tightness	bilateral	nodules /consolidation /atelectasis	positive	negative	TP
29	male	25	fever	bilateral	stripe shadows	negative	negative	TP
30	male	52	cough/chest pain	right	patchy exudation / local consolidation	negative	negative	TP
31	male	21	cough/ sputum/chest tightness	right	patchy exudation	negative	positive	TP

TP, tuberculous pleurisy; NTP, non-tuberculous pleurisy.

### 3.2 Diagnostic accuracy of mNGS and GeneXpert MTB in tuberculous pleurisy

**Figure 2:**
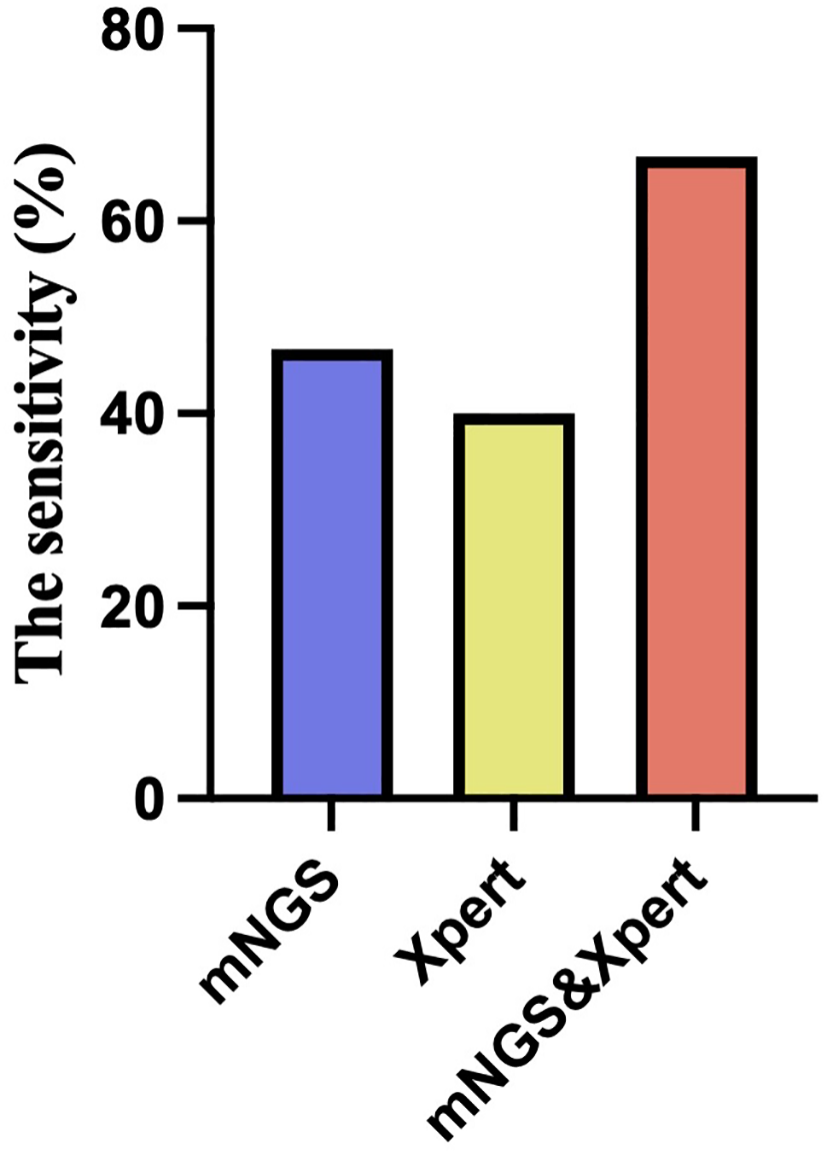
The sensitivity of mNGS, Xpert alone and combined.

Diagnostic accuracy of mNGS and Xpert MTB was evaluated in patients with suspected tuberculous pleurisy using confirmed diagnosis as the gold standard. MTB detection was performed on pleural effusion samples using both methods. The results showed that in the TP group, 7 cases were positive and 8 cases were negative by mNGS, with a sensitivity of 46.67%, specificity of 100%, missed diagnosis rate of 53.33%, misdiagnosis rate of 0%, PPV of 100%, and NPV of 66.67% (**Table 3**; **Figure 2**). Similarly, 6 cases were positive and 9 cases were negative by GeneXpert MTB, with a sensitivity of 40.0%, specificity of 100%, missed diagnosis rate of 60.0%, misdiagnosis rate of 0%, PPV of 100%, and NPV of 64.0% (**Table 3**; **Figure 2**). In the pursuit of robust comparisons between mNGS and Xpert diagnostic performances, the statistical examination was meticulously conducted using the Fisher’s exact probability method, resulting in a bilateral test P value of 1. This outcome indicates the absence of statistical significance, affirming the consistency of the observed data. The results suggest that both mNGS and GeneXpert MTB demonstrate high specificity and PPV, along with comparable sensitivity, for detecting MTB in pleural effusion samples.

**Table 3:** Diagnostic accuracy of the two methods alone and combined detection of mNGS and Xpert for tuberculous pleurisy and non-tuberculous pleurisy (N=31).

value	mNGS	Xpert	mNGS&Xpert
Sensitivity (%)	46.67 (7/15)	40 (6/15)	66.67 (10/15)
Specificity (%)	100 (16/16)	100 (16/16)	100 (16/16)
missed diagnosis (%)	53.33 (8/15)	60 (9/15)	33.33 (5/15)
Misdiagnosed (%)	0 (0/16)	0 (0/16)	0 (0/16)
positive predictive value (%)	100 (7/7)	100 (6/6)	100 (10/10)
negative predictive value (%)	66.67 (16/24)	64 (16/25)	76.19 (16/21)

### 3.3 Diagnostic accuracy of combined mNGS and GeneXpert MTB for tuberculous pleurisy

Further analysis of the diagnostic efficacy of mNGS combined with GeneXpert MTB was performed in patients with suspected tuberculous pleurisy. The results showed that out of the 15 patients with confirmed tuberculous pleurisy, 10 were positive and 5 were negative by the combined test of mNGS and GeneXpert MTB. In contrast, all 16 patients with non-tuberculous pleural effusion were negative by the combined test. The combined test had a sensitivity of 66.66%, specificity of 100%, missed diagnosis rate of 33.33%, and misdiagnosis rate of 0 (**Table 3**; **Figure 2**). The results suggest that the combined test of mNGS and GeneXpert MTB can increase the overall sensitivity of each test and improve the accuracy of the diagnosis.

### 3.4 mNGS analysis reveals microbial profiles of tuberculous and non-tuberculous pleural effusions

**Figure 3:**
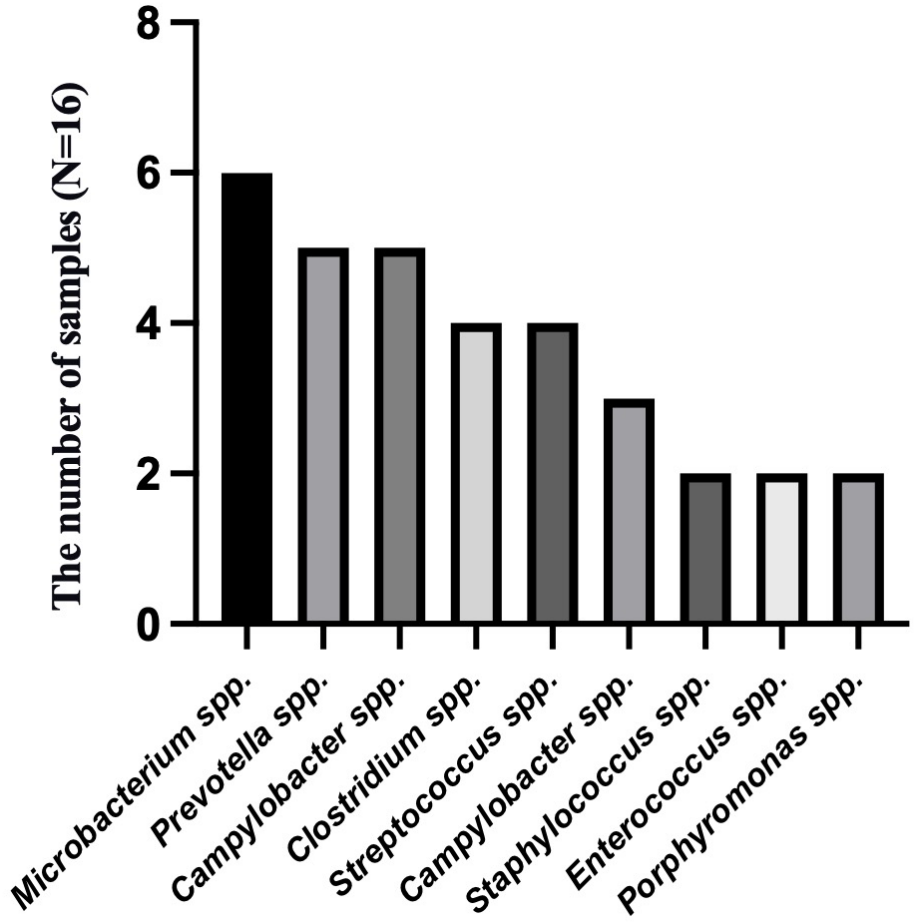
The genus-level identification of the most frequently detected microorganism in the non-tuberculous pleurisy group.

**Figure 4:**
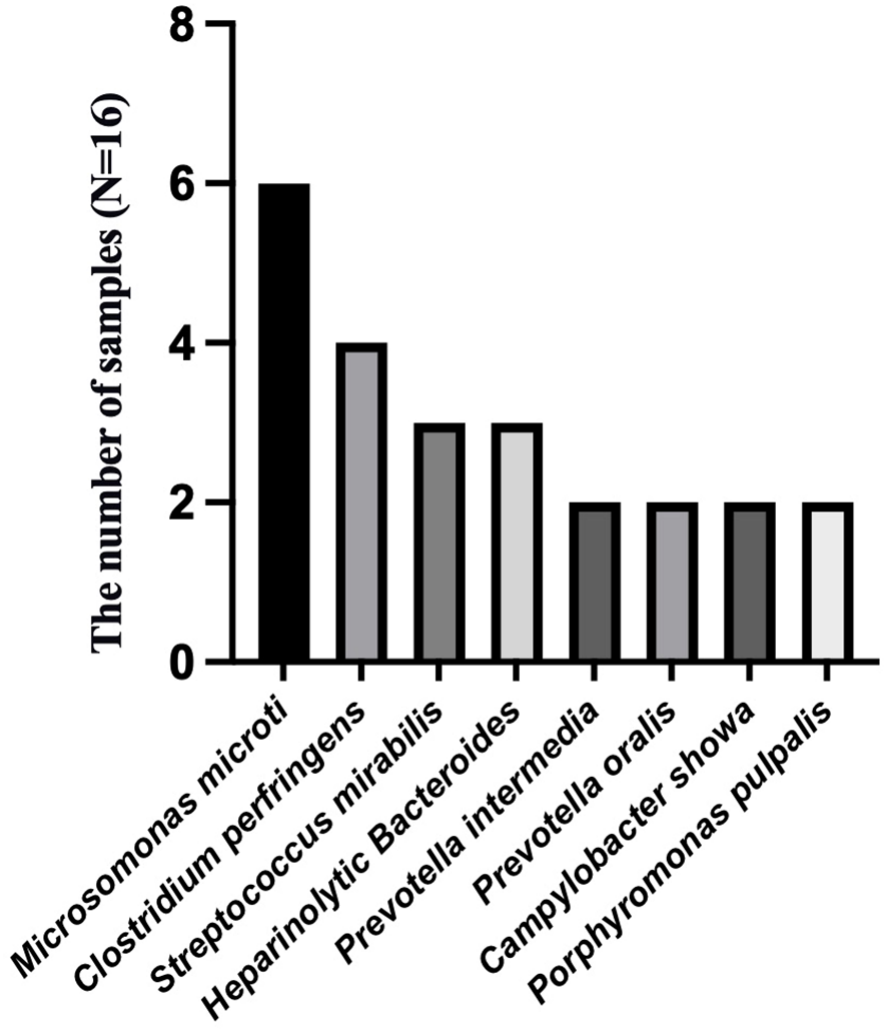
The species-level identification of the most frequently detected microorganism in the non-tuberculous pleurisy group.

To analyze the characteristics of pathogens in patients with tuberculous pleurisy and non-tuberculous pleurisy, we collected mNGS test reports (**Table 4**). The results showed that MTB was detected in 7 out of 15 patients with tuberculous pleurisy, while *Aspergillus fumigatus* was detected in 2 cases. Among the 16 patients with non-tuberculous pleurisy, 6 showed no evidence of any pathogens. One patient was found to have a single pathogen, while 3 patients had two detected pathogens. The remaining 6 patients were found to have multiple (≥ 3) pathogens. In the 10 cases where pathogens were detected, all 10 patients showed the presence of Gram-staining positive organisms, with 8 of them showing the presence of both Gram-staining positive and negative organisms. The most frequently detected genera at the microbial genus level were *Microbacterium* spp. (6/16), *Prevotella* spp. (5/16), *Campylobacter* spp. (5/16), *Clostridium* spp. (4/16), *Streptococcus* spp. (4/16), *Campylobacter* spp. (3/16), *Staphylococcus* spp. (2/16), *Enterococcus* spp. (2/16), and *Porphyromonas* spp. (2/16) (**Figure 3**). The most frequently detected species at the microbial species level were *Microsomonas microti* (6/16), *Clostridium perfringens* (4/16), *Streptococcus mirabilis* (3/16), *Heparinolytic Bacteroides* (3/16), *Prevotella intermedia* (2/16), *Prevotella oralis* (2/16), *Campylobacter showa* (2/16), and *Porphyromonas pulpalis* (2/16) (**Figure 4**). These data suggest that mNGS can detect a diverse range of microbial genera and species in patients with non-tuberculous pleurisy.

**Table 4:** The results of microbial profiles detected by mNGS test.

Case number	The detection result
**1**	Staphylococcus aureus; Bacteroides thetaiotaomicron
**2**	Not detected
**3**	Not detected
**4**	Not detected
**5**	Parvimonas micra; Fusobacterium nucleatum; Fusobacterium nucleatum; Porphyromonas endodontalis; Campylobacter showae; Prevotella denticola; Prevotella denticola
**6**	Parvimonas micra; Bacteroides heparinolyticus
**7**	Not detected
**8**	Staphylococcus epidermidis
**9**	Enterococcus faecalis; Human gammaherpesvirus 4
**10**	Eggerthia catenaformis; Dialister pneumosintes; Parvimonas micra; Bacteroides heparinolyticus; Prevotella intermedia
**11**	Prevotella oris; Parvimonas micra; Streptococcus intermedia; Bacteroides heparinolyticus; Fusobacterium nucleatum
**12**	Fusobacterium nucleatum; Streptococcus milleri; Parvimonas micra; Porphyromonas endodontalis; Prevotella oris
**13**	Parvimonas micra; Prevotella intermedia; Streptococcus milleri; Fusobacterium nucleatum; Campylobacter rectus; Prevotella tannerae
**14**	Not detected
**15**	Enterococcus avium; Bacteroides fragilis; Campylobacter showae; Citrobacter freundii complex
**16**	Not detected
**17**	Mycobacterium tuberculosis complex; Aspergillus fumigatus
**18**	Mycobacterium tuberculosis complex
**19**	Not detected
**20**	Not detected
**21**	Not detected
**22**	Mycobacterium tuberculosis complex
**23**	Not detected
**24**	Not detected
**25**	Mycobacterium tuberculosis complex
**26**	Mycobacterium tuberculosis complex
**27**	Mycobacterium tuberculosis complex
**28**	Mycobacterium tuberculosis complex
**29**	Not detected
**30**	Not detected
**31**	Aspergillus fumigatus

## 4 Discussion

Rapid and accurate diagnosis of tuberculous pleurisy is clinically challenging. This study found that both mNGS and GeneXpert MTB exhibited high specificity and PPV in detecting MTB in pleural effusion samples, while demonstrating comparable sensitivity. The combination of mNGS and GeneXpert MTB improved the sensitivity for the diagnosis of tuberculous pleurisy. Furthermore, mNGS revealed diverse microbial profiles in patients with non-tuberculous pleurisy. The results suggest that mNGS can effectively identify MTB in pleural effusions, potentially contributing to the diagnosis and management of tuberculosis.

In earlier studies, cultures of MTB in pleural effusions were often negative, and tuberculin-induced type 1 helper T cells were found in such samples, leading to the definition of tuberculous pleurisy as a delayed allergic response to MTB or its metabolites (Aktas et al., 2009; Porcel, 2009). However, recent improvements in diagnostic assays have shifted the understanding of the pathogenesis of tuberculous pleurisy, with current evidence suggesting that direct infection with MTB and/or stimulation of the pleura by MTB metabolites causes pleural inflammation (Shaw et al., 2018; Shaw and Koegelenberg, 2021). In our study, MTB was detected in multiple pleural effusion specimens, supporting this current view.

Rapid and precise detection of pathogenic microorganisms is essential for early diagnosis, appropriate medication and accurate prognosis assessments of infectious diseases. Currently, the clinical identification of MTB primarily relies on culture, smear microscopy, and nucleic acid amplification detection. Among these methods, culture is considered the gold standard for identifying MTB and can also be used for drug sensitivity testing. However, culture of pleural fluid with Lowenstein-Jensen medium has a low positive rate (less than 30%) and is time-consuming, which hinders early diagnosis and treatment. Conventional smear microscopy using Ziehl-Nielsen or Auramine stains of pleural fluid also exhibits a low positive rate (less than 10%) and cannot identify specific mycobacterial strains (Gopi et al., 2007; Shaw et al., 2019). Notably, mNGS has emerged as an advanced detection technology capable of directly profiling all nucleic acid sequences (DNA and RNA) from clinical samples using NGS. It can provide results within approximately 24 hours while simultaneously enabling strain identification and detection of drug resistance genes. Furthermore, it enables efficient alignment of the obtained sequences with genomic sequences from various species, thereby determining the types and proportions of microorganisms present in the analyzed samples. mNGS has shown great potential for the early etiological diagnosis of thoracic and abdominal infections due to its high sensitivity and broad pathogenic spectrum detection capabilities (Chen et al., 2021). Huang et al. have demonstrated that mNGS is superior to traditional methods, such as MTB culture and PCR detection, in identifying MTB, bacteria, fungi, and mycoplasma in patients with acute respiratory failure (Huang et al., 2021). In clinical studies, mNGS has been found to be rapid and efficient, with a higher sensitivity (62%) for the diagnosis of extrapulmonary tuberculosis compared to smear (5%), MTB culture (18%), and GeneXpert (31%) (Sun et al., 2021). Additionally, mNGS can detect rare, novel and unknown pathogen sequences, making it even more important for diagnosing difficult and complicated diseases. Studies have also demonstrated the potential of mNGS in the rapid and accurate detection and identification of *Nocardia* species (Ding et al., 2021). By utilizing mNGS, Wilson et al. successfully detected the presence of leptospirosis with central nervous system involvement in a 14-year-old epileptic boy who had experienced recurrent fever of unknown origin (Wilson et al., 2014). Subsequently, the patient received appropriate treatment and achieved complete recovery.

The differential diagnosis of pleural effusion etiology has always been a clinical challenge, particularly for the identification of MTB infection. The clinical utility of the Xpert assay for detecting MTB in sputum is well-established; however, there is a scarcity of studies assessing its diagnostic efficacy in pleural effusion, and the findings are inconclusive. Similar to the study conducted by Du et al., which reported a sensitivity of 43.6% and specificity of 98.6% (Du et al., 2015), our study demonstrated that the Xpert assay exhibited a sensitivity of 40% and specificity of 100% for the detecting MTB in pleural effusions. In contrast, Rufai et al.’s study displayed a higher sensitivity (54.5%) but consistent specificity (100%) with our findings (Rufai et al., 2015). Conversely, both Meldau et al. (Meldau et al., 2014) and Lusiba et al. (Lusiba et al., 2014) found that the sensitivity and specificity of Xpert assay were lower than our study, with the sensitivity was 22.5% and 28.7%, and the specificity was 98% and 96.6%, respectively. The diagnostic performance of the Xpert assay for detecting MTB in pleural effusions demonstrated significant variability, potentially due to regional differences in tuberculosis prevalence and limitations in sample size. Therefore, multi-center and large sample-size studies need to be implemented in the future. Also, there have been few studies investigating the diagnostic efficacy of mNGS for detecting MTB in pleural effusion specimens. Therefore, we conducted this study and evaluated the diagnostic performance of mNGS compared to GeneXpert MTB for the detection of MTB in pleural effusion specimens. Our results demonstrated that both mNGS and GeneXpert MTB exhibited high specificity and PPV in detecting MTB in pleural effusion samples, while demonstrating comparable sensitivity. Although mNGS had a higher positive detection rate of MTB compared to GeneXpert MTB, its cost is considerably higher (Chen et al., 2020). Therefore, a combined approach of mNGS and GeneXpert MTB can be considered in clinical settings where resources allow. Previous studies have also investigated the use of combined tests for the diagnosis of tuberculous pleural effusion. For example, a study by Xu et al. found that combining ADA and interferon-γ release assay tests had a sensitivity of 96.9% and a specificity of 87.5% for diagnosing tuberculous pleural effusion (Xu et al., 2017). A systematic review and meta-analysis showed that the combined test of sTREM-1 and ADA had a sensitivity of 90.4% and a specificity of 82.4% for diagnosing tuberculous pleurisy (Liang et al., 2008). Compared to these previous studies, our study showed that the combined test of mNGS and GeneXpert MTB had a slightly lower sensitivity (66.67%) but a higher specificity (100%) for diagnosing tuberculous pleural effusion. The high specificity of the combined test indicates that it has a low false positive rate and can effectively distinguish tuberculous from non-tuberculous pleural effusion. However, the relatively low sensitivity suggests that the combined test may miss some cases of tuberculous pleural effusion. Therefore, it may be necessary to combine the combined test with other diagnostic methods to improve its diagnostic accuracy. In addition, mNGS had the advantage of detecting a wide range of microbial profiles in tuberculous and non-tuberculous pleural effusion, which can aid in the differential diagnosis of pleural effusion etiology. In this study, two cases of concurrent Aspergillus infection were identified among patients with tuberculous pleurisy. Among patients with non-tuberculous pleurisy, the most frequently detected microbial genera included Microbacterium spp. (6/16), Prevotella spp. (5/16), Campylobacter spp. (5/16), Clostridium spp. (4/16), and Streptococcus spp. (4/16). The most commonly detected microbial species were Microsomonas microti (6/16), Clostridium perfringens (4/16), Streptococcus mirabilis (3/16), and Heparinolytic Bacteroides (3/16). These findings significantly contribute to accurate treatment and provide valuable reference for studying the microbiome of pleural fluid samples. Furthermore, a limited presence of other microbial species was observed in patients with tuberculous pleurisy, with Aspergillus fumigatus being detected in only two individuals. However, it was found that six patients with non-tuberculous pleurisy exhibited a higher prevalence (≥3) of multiple microorganisms. The findings suggest a decrease in microbial diversity and an impairment of microbiome structure in patients with tuberculous pleurisy. Further investigation is warranted to elucidate the underlying mechanism behind this intriguing observation.

According to statistics, approximately 15% to 20% of cases remain etiologically unidentified despite undergoing a series of conventional diagnostic methods. Moreover, certain specific populations, including elderly individuals and those with compromised overall health or inability to undergo lung biopsy and bronchoscopy procedures for obtaining pathological and bronchoalveolar lavage fluid specimens, pose additional challenges. Given the easy retention of pleural fluid samples, it is of great value and advantage to perform Xpert assay and mNGS to assist in the diagnosis of tuberculous pleurisy, particularly in this specific population. Nevertheless, it is important to acknowledge that mNGS still represents a significant advancement in pathogen detection. Specifically, the vast majority of nucleic acid sequence reads (typically exceeding 99%) in patient samples originate from human hosts, thereby limiting the comprehensive analytical sensitivity of pathogen detection methods. Additionally, due to the complex nature of clinical samples, there may be a scarcity of available pathogenic information leading to data loss or confusion between pathogen data and normal flora that are challenging to differentiate. Furthermore, potential microbial contamination during sampling, reagent handling, and laboratory environment can adversely impact pathogenic identification by overestimating microbial diversity within samples and consequently complicating result analysis and interpretation.

The mNGS technology encompasses a diverse range of disciplines and technical components, including sample collection and pretreatment, microbial nucleic acid extraction, library preparation, high-throughput sequencing, bioinformatics analysis, and professional interpretation of detection reports. In comparison to commonly employed methods in clinical diagnosis such as microbial culture, smear microscopy, and PCR which incur relatively lower costs ranging from a few dollars to tens of dollars per sample analysis, the utilization of mNGS involves additional expenses due to factors like training skilled technicians, utilizing expensive instruments, operating and maintaining equipment as well as implementing quality control measures. Consequently, the total expenses for each sample analysis may amount to hundreds or even thousands of dollars. However, advancements in automation and standardization of mNGS protocols are expected to lead to a significant reduction in costs facilitating its widespread adoption in clinical settings for rapid and precise diagnosis.

Our study still has several limitations. First, the relatively smaller sample size of the present study may introduce potential bias in the obtained results. In future investigations, we aim to address this limitation by expanding our sample size and conducting more comprehensive and in-depth research on mNGS in various forms of tuberculosis, including pulmonary tuberculosis as well as extrapulmonary manifestations such as tuberculous pleurisy, tuberculous meningitis, and tuberculous peritonitis. Additionally, to enhance the reliability of our findings for guiding clinical applications, we plan to perform a comprehensive analysis on a broader range of indicators including immunological markers, ADA levels, Mycobacterium tuberculosis culture results, and smear microscopy outcomes through multi-regional and multi-center sampling.

In conclusion, this study suggests that mNGS can effectively identify MTB in pleural effusions and offers the advantage of detecting a wide range of microbial profiles in both tuberculous and non-tuberculous pleural effusions. These findings have the potential to significantly impact the diagnosis and treatment of patients with pleural effusion, ultimately leading to better patient outcomes.

## Data availability statement

The original contributions presented in the study are included in the article/supplementary material. Further inquiries can be directed to the corresponding author.

## Ethics statement

The studies involving humans were approved by the Ethics Committee of The First Affiliated Hospital of Zhengzhou University. The studies were conducted in accordance with the local legislation and institutional requirements. The participants provided their written informed consent to participate in this study.

## Author contributions

FH, HW and LM carried out the studies, participated in collecting data, and drafted the manuscript. QP and CZ participated in its design and collecting data. FH, RQ and HW performed the acquisition, statistical analysis and participated in its design. All authors contributed to the article and approved the submitted version.
